# Evidence base for the management of flexor tendon injuries

**DOI:** 10.1016/j.amsu.2020.05.023

**Published:** 2020-06-01

**Authors:** Athanasius Ishak, Akshaya Rajangam, Ankur Khajuria

**Affiliations:** a Faculty of Life Sciences and Medicine, Centre for Human and Applied Physiological Science (CHAPS), King's College London, Strand, London, WC2R 2LS, UK; b Department of Plastic Surgery, St Thomas' Hospital, London, SE1 7EH, UK; c Kellogg College, University of Oxford, Oxford, UK

Dear Editor,

We appreciate the comments by the authors in their letter to the editor on our published article [1].

The points raised are important and expand on our review. Venting critical annular pulleys enables flexor tendons to glide more freely which in turn has benefits for functional recovery. Partial venting of the A2 pulley or the entire A4 pulley, when necessary, is advocated and can improve outcomes. In most cases part of the A2 pulley is preserved and venting up to two-thirds of the A2 pulley is considered safe provided that the other pulleys are intact [2,3]. In our review we thought It important to stress that venting must be done meticulously and limited to only the extent necessary. Interestingly, there are publications of clinical outcomes following release of the entire A2 pulley which report no clinically evident bowstringing [4,5]. However, these findings are limited by small cohort numbers and more robust clinical studies are needed to evaluate this practise.

Regarding venting of the A4 pulley, we agree that absolute preservation is not always necessary and should not be adhered to as dogma. This is particularly true when all other pulleys are intact.

Effective and safe rehabilitation protocols are of paramount importance to restoring digital motion and function after flexor tendon repair surgery. A balance must be achieved between the rigor of the rehabilitation to reduce adhesion formation and the risk of tendon rupture. As the authors correctly point out in their letter, early active mobilisation protocols are commonly used for post-operative rehabilitation and have largely replaced passive motion regimens. Although effective post-operative rehabilitation protocols are crucial to ensuring the best clinical outcomes this was beyond the scope of our review which aimed to summarise the clinical evidence base for primary flexor tendon repair techniques at each anatomical zone. We would like to thank the authors for emphasising this important aspect of flexor tendon repair in their letter.

## Ethical approval

Not applicable.

## Sources of funding

There are no sources of funding to declare.

## Author contribution

Writing the paper: Athanasius Ishak, Akshaya Rajangam and Ankur Khajuria.

## Consent

Not applicable.

## Guarantor

Athanasius Ishak

## Declaration of competing interest

The authors have no conflicts of interest to declare.
